# Research progress of circRNA as a biomarker of osteoporosis

**DOI:** 10.3389/fgene.2024.1378026

**Published:** 2024-05-09

**Authors:** Xing Du, Zhongyao Chen, Wei Shui

**Affiliations:** ^1^ Department of Orthopedics, The First Affiliated Hospital of Chongqing Medical University, Chongqing, China; ^2^ Orthopedic Laboratory of Chongqing Medical University, Chongqing, China

**Keywords:** osteoporosis, biomarkers, osteoblasts, osteoclasts, circRNA

## Abstract

Osteoporosis, as a chronic metabolic bone disease, has the characteristic of insidious disease progression, which often leads to relatively delayed disease diagnosis. Therefore, early screening for osteoporosis has become a major public health challenge. The latest research indicates that circRNA is widely involved in the regulation of bone metabolism and is closely related to the occurrence and development of osteoporosis. Based on its high degree of sequence conservation and stability, circRNA has the potential to become a new clinical biomarker. The study of biomarkers is generally based on body fluid samples or adjacent tissue samples, with blood being the most commonly used, which can be divided into sources such as serum, plasma, peripheral blood monocytes, and plasma exosomes. Therefore, this article aims to review the research status of circRNA as a biomarker of osteoporosis.

## 1 Background

Osteoporosis is a common systemic metabolic disorder in elderly people, especially postmenopausal women, characterized by microstructural degeneration of bone tissue, decreased bone mass per unit volume, and increased bone fragility (Lizneva et al., 2018; Compston et al., 2019). With the accelerated aging of the population, the incidence rate of osteoporosis is also getting higher and higher. Most patients get a clear diagnosis after the occurrence of osteoporosis fractures, which makes the diagnosis of osteoporosis lag behind to varying degrees, becoming a public health problem that seriously endangers health, and bringing a heavy burden to patients and society (Feng et al., 2018; El et al., 2019).

At present, the diagnosis of osteoporosis in clinical practice is mainly achieved through bone mineral density (BMD) testing, but slow changes in bone density have certain limitations and lag. Biochemical indicators related to bone metabolism can reflect the state of bone metabolism and serve as auxiliary diagnostic and differential diagnostic indicators, but their sensitivity in the early stages of disease development is still insufficient. Therefore, actively discovering and exploring potential biomarkers related to early diagnosis of osteoporosis, early intervention to prevent the occurrence of serious complications of osteoporosis, has important clinical value and social significance.

Circular RNA (circRNA) is a group of non-coding RNAs with strong stability and tissue specificity, playing an important role in cellular function. With the deepening of research on epigenetics, a large number of studies have shown that circRNA participates in regulating the differentiation and function of osteoblast (OB) and osteoclast (OC) through various pathways, thereby affecting bone formation and absorption, closely related to the occurrence and development of osteoporosis, and has the potential as a clinical biomarker of osteoporosis (Yang et al., 2020; Chen et al., 2021).

In summary, this article intends to review the role of circRNA in bone metabolism through literature review, as well as the research progress of circRNA as a biomarker in the early diagnosis of osteoporosis.

## 2 Biomarkers of osteoporosis

Biomarkers refer to indicators that can be objectively measured and evaluated, reflect physiological or pathological processes, and have biological effects on exposure or treatment interventions. Biomarkers not only explore the pathogenesis at the molecular level, but also have unique advantages in accurately and sensitively evaluating early and low-level damage, providing early warning and largely providing auxiliary diagnostic basis for clinical doctors.

Until now, clinical technology has developed and bone density remains the main indicator for diagnosing osteoporosis, predicting the risk of osteoporotic fractures, and evaluating the efficacy of anti osteoporosis drugs. However, due to the need for dual energy DXA instruments to measure bone density, the change process is slow and cannot reflect the short-term changes in bone density. Therefore, dynamic monitoring and observation often need to be combined with bone metabolism indicators (Wang et al., 2017). Different biomarkers play corresponding roles in the diagnosis of osteoporosis, and the dynamic state of bone metabolism can be reflected by bone metabolism biomarkers. circRNA has been found to play a potential role in early diagnosis of osteoporosis.

Bone homeostasis refers to the dynamic equilibrium process between new bone formation and old bone resorption in the skeletal system, which is essential for maintaining bone health and mineral homeostasis in the body (Ensrud and Crandall, 2017). It is achieved primarily through the interaction between osteoblasts and osteoclasts, which are responsible for bone formation and bone resorption, respectively. When this balance is broken, it may lead to bone disorders such as osteoporosis (Ensrud and Crandall, 2017). Osteoblasts play a crucial role in the process of bone formation, and bone alkaline phosphatase (B-ALP) and osteocalcin can reflect the activity of osteoblasts and directly reflect the state of bone formation. At the same time, type I collagen synthesized by osteoblasts can decompose and release procollagen type I amino-terminal propeptide (PINP) and procollagen type I carboxy-terminal propeptide (PICP), which can also serve as biomarkers reflecting the state of bone formation. Sclerostin, as a specific expression protein of osteoblasts, can serve as a biomarker for regulating bone formation. Osteoprotegerin (OPG) is a novel biomarker of bone formation which can inhibit the differentiation of osteoclast precursor cells into mature osteoclasts, thereby reducing bone resorption and increasing bone formation.

The metabolites of bone resorption and substances that reflect osteoclast activity are commonly used as biomarkers of bone resorption. Tartrated resistant acid phosphatase (TRAP), type I collagen cross-linked C-terminal peptide (CTX), type I collagen cross-linked N-terminal peptide (NTX), pyridinoline (PYD), and deoxypyridinoline (DPD), can serve as biomarkers reflecting bone resorption status for they are associated with osteoclast activity. Hydroxyproline (HOP) and cathepsin K (Cat K), as metabolites of bone resorption, can also serve as biomarkers of bone resorption. However, HOP can originate from other tissues such as the skin, thus lacking specificity.

The existing bone metabolism biomarkers (Table 1) are mostly biochemical products of bone metabolism, which can be detected from body fluids and have the characteristics of safety, convenience, speed, and economy. However, these bone metabolism biomarkers can generally only reflect the current status of bone metabolism from a single perspective and can only be used for monitoring bone balance, and cannot be directly used for the diagnosis of osteoporosis (Eastell and Szulc, 2017). Therefore, searching for more specific and sensitive biomarkers to play a greater role in the early diagnosis of osteoporosis is the focus of future research.

**TABLE 1:** Biomarkers of bone metabolism in bone formation and absorption.

Category	Name	Source	Function
Bone formation	B-ALP	OB	Positive correlation with OB activity (high specificity)
	PINP/PICP	Type I Pro-collagen of OB	Positive correlation with bone formation status (high specificity)
	Osteocalcin	Mature OB	Positive correlation with OB activity (high specificity)
	Sclerostin	OB	Negative correlation with bone formation status (high specificity)
	OPG	OB	Inhibit the differentiation of OC precursors, reduce bone resorption, and increase bone formation
Bone resorption	TRAP	OC	Positive correlation with OC activity and bone resorption status
	CTX/NTX	Type I collagen	Positive correlation with bone resorption status
	PYD/DYD	Type I collagen	Positive correlation with bone resorption status
	HOP	Bone collagen, skin, complement	Reflect the degree of bone resorption (low specificity)
	Cat K	OC	Positive correlation with bone resorption status

B- ALP, bone alkaline phosphatase; PINP, procollagen type I amino-terminal propeptide; PICP, procollagen type I carboxy-terminal propeptide; OPG, osteoprotegerin; TRAP, tartrated resistant acid phosphatase; CTX, type I collagen cross-linked C-terminal peptide; NTX, type I collagen cross-linked N-terminal peptide; PYD, pyridinoline; DPD, deoxypyridinoline; HOP, hydroxyproline; Cat K, cathepsin K; OB, osteoblast; OC, osteoclast.

## 3 CircRNA as a biomarker of osteoporosis

CircRNA, as an non-coding RNA with a stable circular structure, is stably present in blood and various bodily fluids, and can regulate the occurrence and development of osteoporosis at the genetic level. Therefore, it has the potential to become a biomarker for early diagnosis of osteoporosis (Zhang et al., 2018).

### 3.1 Biological characteristics and functions of circRNA

As a hot research topic in the field of non-coding RNA, circRNA can be expressed in various organisms with a stable circular structure, which can prevent degradation mediated by exonucleases, making it a special molecular marker in diseases (Han et al., 2018; Zhao et al., 2018; Kristensen et al., 2019). Research has shown that circRNA can play important regulatory roles in the occurrence, development, and evolution of diseases through different mechanisms (Liang et al., 2021; Liu and Chen, 2022; Wu et al., 2022; Zhang et al., 2023). At present, a large number of related studies have confirmed that circRNA plays an important role in the occurrence, development, and treatment of neurological, cardiovascular, and tumor diseases. It can serve as an important biomarker for disease diagnosis and prognosis judgment, as well as a molecular target for treatment. Therefore, circRNA has very important research significance and clinical application value (Li et al., 2018; Ma et al., 2020; Peng et al., 2022).

### 3.2 CircRNA regulates osteoblasts and bone formation

Osteoblasts, as important cells in the process of bone formation, can regulate the bone formation process through various aspects such as bone mineralization, secretion of bioactive factors, and participation in bone matrix synthesis.

For the differentiation activity of osteoblasts, Qian et al. (2017) detected differential expression of circRNA during BMP-2 induced differentiation of mouse osteoblasts using RNA sequencing technology and they found that the expression level of circRNA_19142, circ_5846, and circle_10042 were significantly increased in highly differentiated osteoblasts. And through experiments, it was found that BMP2 passes through the circ_19142/circ_5846 targeting miRN-mRNA axis induces osteogenic differentiation in MC3T3-E1 cells. However, in the study of the mechanism by which YAP1 promotes osteogenic differentiation of bone mesenchymal stem cells (BMSCs) and MC3T3-E1, Huang et al. (2020) found that circle_0024097 can be achieved through miR376b-3p/YAP1 axis and Wnt/β-catenin pathway promotes osteogenic differentiation of BMSCs and MC3T3-E1 to promote bone formation.

In the study of growth factors related to osteogenic differentiation, osteopontin (OPN) and collagen protein α-1 (COL15A1) had been found to regulate the differentiation and mineralization of MC3T3-E1 and MDPC23 cells, and this process is mediated by circ_003795 through the miR-1249-5p/COL15A1 axis (Wu et al., 2020). There was also experimental study indicating that silencing the circle_009056 can increase the expression of miR-22-3p and reduce the expression levels of BMP7 and Runx2 (Wu et al., 2018). Therefore, circle_009056 can serve as a sponge for miR-22-3p, regulating the osteogenic process induced by calcitonin gene related peptides in cells. In the study of circle_0062582, there were different results between different researches. Li et al. (2021) found that circle_0062582 can promote osteogenic differentiation of BMSCs by targeting the miR-145/CBFB axis, and upregulate the levels of osteogenic differentiation related proteins such as OSX, OCN, and COL1. However, Chen et al. (2022) found that circle_0062582 upregulate the expression of Smad5 through sponge like miR-197-3p, thereby promoting the proliferation and osteogenic differentiation of BMSCs. Through different methods of studying and analyzing different types of osteoblasts, it was found that circRNA can reflect the regulatory effects of osteoblasts on differentiation, proliferation, and other aspects at different stages of bone formation.

### 3.3 CircRNA regulates osteoclasts and bone resorption

With the continuous deepening of research on circRNA, more and more studies have shown that circRNA participates in the maturation, differentiation, apoptosis and other processes of osteoclasts through different mechanisms. In the study of osteoclast proliferation, Miao et al. (2020) found that circRNA_009934, as the ceRNA of miR-5107, regulates the expression of its downstream target gene TRAF6 and promotes osteoclast generation. In the study of osteoclast differentiation, Chen et al. (2019) found the differential expression of circ_28313 in the process of bone marrow mononuclear macrophage (BMM) differentiation through microarray analysis; meanwhile, bioinformatics analysis indicates that circle_28313, miR-195a, and CSF1 form a ceRNA network, circ_28313 indirectly regulates the expression of the target gene CSF1 through miR-195a, thereby regulating the differentiation of BMM cells into osteoclasts. Liu et al. (2020) revealed the interaction mechanism of circ_Hmbox1/miR-1247-5p/Bcl6 through bioinformatics analysis and luciferase experiments. Circle_Hmbox1 mainly inhibits the RANKL induced osteoclast differentiation by binding to miR1247-5p, and miR-1247-5p can target Bcl6 to regulate osteoclast differentiation and osteoblast differentiation, participating in the regulation of bone metabolism. It was also discovered that circle_0007059 was upregulated in both osteoporosis patients and human bone marrow stromal cells during their osteoclastogenesis, circ_0007059 can inhibit the differentiation of BMSCs into osteoclasts through the miR-378/BMP-2 axis (Liu et al., 2021).

## 4 CircRNA in early diagnosis of osteoporosis

At present, the diagnosis of osteoporosis is still mainly based on bone density, but due to its limitations and inconveniences, it often cannot be used for early diagnosis of osteoporosis. CircRNA can stably and widely exist in human body fluids, blood, and other tissue specimens, and its expression levels vary at different stages and tissues, with temporal and tissue specificity. These advantages make circRNA a hot topic in current research (Li, 2023). Many clinical and experimental studies have confirmed that there are significant differences in the expression of circRNA in the peripheral blood, bone marrow stem cells, or bone tissue of patients with osteoporosis (Table 2). Therefore, circRNA has the potential to serve as an early diagnostic biomarker of osteoporosis.

**TABLE 2:** The mechanism and potential of circRNA as a biomarker for osteoporosis in different samples.

Specimens	Researcher	Method	circRNA	Mechanism	Diagnostic value (ROC curve)
Serum and plasma	Huang et al. (2019)	Case-control study	circ_0006873	N/R	N/R
	Huang et al. (2019)	Case-control study	circ_0002060	N/R	AUC = 0.746, *p* < 0.05, sensitivity = 78%, specificity = 69%
	Han et al. (2020)	Case-control study	circ_0076690	circ_0076690 sponges miR-152 to promote the expression of Runx2 and regulate the osteogenic differentiation of hBMSCs	AUC = 0.8299, *p* < 0.001, sensitivity = 79%, specificity = 85%
PBMCs	Guan et al. (2021)	Case-control study	circ_0021739	Overexpression of circ_0021739 reduces the expression of miR-502-5p and inhibits the differentiation of OCs	AUC = 0.849, *p* < 0.001, sensitivity = 100%, specificity = 42.9%
	Zhao et al. (2018)	Case-control study	circ_0001275	N/R	AUC = 0.849, *p* < 0.001
Serum exosomes	Zhi et al. (2021)	Case-control study	circ_0006859	circ_0006859 sponges miR-431-5p to upregulate ROCK1 and inhibit the osteogenic differentiation	AUC = 0.8974, *p* < 0.001, sensitivity = 93.1%, specificity = 93.33%
	Qiao et al. (2020)	Case-control study	circ_0048211	circ_0048211 negatively targets miR-935p to upregulate BMP2 and promote bone differentiation	N/R
	Wen et al. (2020)	Case-control study and *in vitro* cell experiment	circ_0076906	circ_0076906 sponges miR-1305 to upregulate the expression of OGN and induce the osteogenic differentiation of hBMSCs	N/R
BMSCs	Yu and Liu (2019)	Case-control study and *in vitro* cell experiment	circ_0016624	circ_0016624 sponges miR-98 to enhance the expression of BMP2 and promote bone formation	N/R
	Liu et al. (2021)	Case-control study and *in vitro* cell experiment	circ_0007059	circ_0007059 targets miR-378 to inhibit the osteoclastic differentiation of BMSCs	N/R
	Ji et al. (2021)	Case-control study and *in vitro* cell experiment	circ_0006215	circ_0006215 targets miR-9425p to regulate the expression of Runx2 and VEGF in BMSCs, promoting osteogenic differentiation and angiogenesis	N/R

BMSC, bone mesenchymal stem cell; Runx2, Runt-related transcription factor 2; PBMC, peripheral blood mononuclear cell; OC, osteoclast; ROCK1, Rho-associated, coiled-coil-containing protein kinase; BMP2, bone morphogenetic protein 2; OGN, osteoglycin; VEGF, vascular endothelial growth factor; ROC, receiver operator characteristic curve; AUC, area under curve; N/R, not reported.

### 4.1 CircRNA in serum and plasma

Serum and plasma, as important components of blood, contain various molecular components such as proteins, lipids, vitamins, inorganic substances, nucleic acid derivatives, etc. As a commonly used clinical test specimen, it has the advantages of easy acquisition and stable quality, and has also been widely used in the study of circRNA in recent years. Yao et al. (2020) performed circRNA sequencing on the serum of 20 osteoporosis patients with vertebral compression fractures, and detected a total of 884 differentially expressed circRNA, of which 554 were upregulated and 330 were downregulated. Huang et al. (2019) found that the expression level of circle_0006873 and circle_0002060 were both significantly elevated in serum of elderly patients with osteoporosis, and significantly correlated with BMD and T values. ROC curve displayed that circle_0002060 (AUC = 0.746, *p* < 0.05, sensitivity = 78%, specificity = 69%) had the potential to be further developed as a biomarker of osteoporosis. Han et al. (2020) found that circle_ 0076690 had the diagnostic value for osteoporosis (ROC curve displayed the AUC = 0.8299, *p* < 0.001, sensitivity = 79%, specificity = 85%), and through bioinformatics analysis and further experiments, it was also found that circ_0076690 can regulate the osteogenic differentiation of BMSCs by sponging miR-152 and targeting Runx2. Therefore, circle_0076690 can affect bone metabolism by regulating osteogenic differentiation, and is therefore considered a potential biomarker for the diagnosis of osteoporosis.

### 4.2 CircRNA in peripheral blood monocytes

As precursor cells of osteoclasts, peripheral blood mononuclear cells (PBMCs) can be induced to differentiate into osteoclast like cells, thus PBMCs had close relationship with osteoporosis. Besides, the expression level of monocyte RNA in peripheral blood circulation is stable. Therefore, circRNA in PBMC has good potential to become a biomarker of osteoporosis. Through chip sequencing and qPCR, Guan et al. (2021) found that the expression of circle_0021739 was significantly increased in PBMC of postmenopausal osteoporosis patients (PMOP), and ROC analysis showed (AUC = 0.849, *p* < 0.001, sensitivity = 100%, specificity = 42.9%) tha circle_0021739 has good potential as a biomarker for diagnosing PMOP. Further research had found that circle_0021739 can target miR-502-5p to regulate osteoclastogenesis, providing a new approach for the development of early prevention and diagnosis methods for osteoporosis. Zhao et al. (2018) studied the PBMC from 58 PMOP patients and 41 postmenopausal elderly healthy volunteers, and found that there was a significant difference in the expression of circ_0001275, and the expression level was negatively correlated with T value. ROC curve displayed that circle_0001275 had high diagnostic value (AUC = 0.759, *p* < 0.001) and can serve as a potential biomarker for PMOP.

### 4.3 CircRNA in serum exosomes

Exosomes are disc-shaped vesicles with a diameter of 30–150 nm, carrying a large amount of biological information such as proteins, nucleic acids, and transcription factors, and can exert regulatory effects after entering target cells. In addition, the lipid bilayer of exosomes can protect RNAs from degradation by ribonucleases, thereby preserving their functional activity and facilitating the collection and storage of marker samples. Zhi et al. (2021) found the expression of circ_0006859 in serum exosomes of osteoporosis patients was upregulated than the healthy control group, and ROC curve showed that the circle_0006859 can distinguish patients with osteoporosis from the healthy control group (AUC = 0.8974, *p* < 0.0001, sensitivity = 93.1%, specificity = 93.33%). Further bioinformatics analysis and experiments had found that circ_0006859 can upregulate ROCK1, inhibit bone formation, and promote fat formation by sponge forming miR-431-5p, and finally leading to the occurrence of osteoporosis. Moreover, after 9 months of anti-osteoporosis treatment, the expression level of circ_0006859 in serum exosomes was also significantly downregulated, which further confirmed the enormous potential of circ_0006859 as a biomarker of osteoporosis.

### 4.4 CircRNA in BMSCs and bone tissue

BMSCs, as important cells in bone metabolism, can promote bone repair and development, and are closely related to the occurrence and development of osteoporosis. Many circRNA in BMSCs can serve as potential new targets for the treatment of osteoporosis. In the study of BMSCs in osteoporosis patients, Qiao et al. (2020) found that the expression of circle_0048211 was negatively correlated with miR-93-5p and positively correlated with BMP2. Circle_0048211 can negatively target miR-93-5p and upregulate BMP2, thereby delaying the progression of PMOP. Through the study of bone tissue specimens from osteoporosis patients, Wen et al. (2020) found that circ_0076906 upregulateed the expression of osteoglycin (OGN) through sponge forming miR-1305 to promote osteogenic differentiation of BMSCs and ultimately slowed down the development of osteoporosis. Yu and Liu (2019) found that circle_0016624 can improve bone metabolism and prevent the occurrence of osteoporosis by enhancing the expression of BMP-2 and the generation of osteogenic inducing factors through sponge forming miR-98. Liu et al. (2021) demonstrated that circle_0007059 can directly target miR-378, which in turn targets BMP-2 and overexpression of circs_0007059 can inhibit the differentiation of BMSCs into osteoclasts. Therefore, targeting the circle_0007059/miR-378/BMP-2 axis may be a new approach for treating osteoporosis. Ji et al. (2021) reported a new finding that circle_0006215 can regulate the expression of Runx2 and VEGF in BMSCs by competitively binding to miR-942-5p, thereby promoting osteogenic differentiation and angiogenesis. At the same time, in the cortical bone defect model, circ_0006215 was also validated for its role in promoting bone repair. Therefore, circle_0006215 has enormous potential to become a new target for the treatment of elderly osteoporosis.

## 5 Conclusion

In summary, many circRNA have been proven to regulate bone metabolism through various mechanisms, affecting the proliferation, differentiation, and apoptosis of osteoblasts, osteoclasts, and BMSCs, as well as regulating the processes of bone formation and resorption. CircRNA, due to its unique and stable circular structure, as well as its spatiotemporal specificity and tissue/cell specificity, can be stably expressed in serum, cells, exosomes, and tissues. It has gradually shown excellent potential as a diagnostic biomarker of osteoporosis.

At present, in the study of exploring a circRNA as biomarker of osteoporosis, the candidate circRNA were preliminary screened by using microarray or circRNA sequencing technology to analyze the differentially expressed circRNA in clinical samples of osteoporosis, and the selected circRNA were finally confirmed via ROC analysis. Subsequently, the reliability and sensitivity of circRNA as a biomarker will be verified through experiments, and bioinformatics and transcriptomics studies can also be used to analyze and demonstrate its mechanism in the occurrence and development of osteoporosis. However, according to previous research reports, circRNA cause dysregulation of bone metabolism by forming a ceRNA network of circRNA/miRNA/mRNA, thereby affecting the progression of osteoporosis, but its precise targets are often difficult to find. With the development of bioinformatics and gene sequencing technology, research on circRNA as a diagnostic marker in osteoporosis will become more and more in-depth.

As a biomarker, it must have characteristics such as stable properties, good reproducibility, convenient collection, and easy repeated detection for dynamic observation of its expression level. In previous research, different tissue samples from the same source often yield different research results. Therefore, the source, processing, preservation, and measurement methods of samples are particularly important. As specimens, serum, peripheral blood monocytes, and serum exosomes all have the above advantages, and subsequent preservation and processing are also relatively simple. How to select appropriate categories from these samples as early diagnostic biomarkers for osteoporosis is an urgent issue that needs attention.

Meanwhile, due to the small sample size, there are significant differences in research conclusions. In the future, with the development of transcriptomics, genomics, and imaging, the combined application of multiple biological diagnostic biomarkers, such as the combination of humoral and imaging biomarkers, will become available. In addition, further *in vivo* experimental verification and follow-up observation of circRNA with enormous potential as a biomarker can be carried out through multi-center large-scale population studies, in order to achieve the clinical application of circRNA as a diagnostic biomarker in osteoporosis.
